# High-Efficiency Wireless-Power-Transfer System Using Fully Rollable Tx/Rx Coils and Metasurface Screen

**DOI:** 10.3390/s23041972

**Published:** 2023-02-10

**Authors:** Woosol Lee, Yong-Kyu Yoon

**Affiliations:** Electrical and Computer Engineering Department, University of Florida, Gainesville, FL 32611, USA

**Keywords:** metasurface, metamaterial, wireless power transfer (WPT), efficiency enhancement, misalignment, negative refraction property, beam focusing, fully rollable WPT system

## Abstract

This work presents a high-efficiency reconfigurable wireless-power-transfer (WPT) system using fully rollable Tx/Rx coils and a metasurface (MS) screen working at 6.78 MHz, for the first time. The MS screens are placed between the Tx and Rx to magnify the power-transfer efficiency (PTE) of the WPT system. The proposed MS-based WPT can be rolled down or rolled up as required, which allows end-users to use the space more flexibly. In the measurement results, the PTE of the WPT is improved from 13.32% to 32.49% at a power-transfer distance (PTD) of 40 cm with one MS screen, 5.42% to 42.25% at a PTD of 50 cm with two MS screens, 1.78% to 49% at a PTD of 60 cm with three MS screens, 0.85% to 46.24% at a PTD of 70 cm with four MS screens. The measured PTE results indicate that the demonstrated MS screens are greatly effective for magnifying the PTE and the PTD of the WPT. In addition, the measured PTE results in the misaligned condition verify that the MS screens also help increase the PTE of the WPT even in the misalignment condition.

## 1. Introduction

With the recent advances in the area of wireless power transfer (WPT), there has been an ever-growing demand for high power-transfer efficiency (PTE) of WPT and a long power-transfer distance (PTD) for end users. Currently, most WPT systems have adopted wireless power transfer via inductive magnetic field coupling between non-resonating coils. However, due to the limited transfer distance of inductive-coupling-based WPT systems, the usability of the current WPT systems is significantly hampered. Alternatively, the magnetic resonance coupling technique can be used, as this can expand power-transmission capabilities to mid-range or long-range distances (i.e., more than three times larger than the radius of the coil [1]). However, as the PTD of the magnetic-coupling-based WPT systems increases, the coupling between the resonating transmitter (Tx) and receiver (Rx) gradually diminishes, resulting in degraded PTE and limited PTD in WPT systems [2].

Researchers have reported that metamaterials (MTMs) or metasurfaces (MS, two-dimensional MTMs) could be used to boost the PTE and PTD of WPT systems [3]. MTMs are artificially engineered materials that exhibit unusual electromagnetic (EM) properties, such as evanescent wave amplification and negative refraction, thereby being able to increase the PTE [4]. In general, MTMs are exploited to magnify the PTE of the WPT by placing one or more MTMs between Tx and Rx. However, a concern is that some previously reported MTMs have had bulky and volumetric structures such as 3D and thick. printed circuit-board-based structures, reducing the practicality of MTM slabs [5,6,7,8,9,10,11,12]. If additional volumetric MTM slabs were permanently located in the power-transfer path, it would greatly limit the usage of the space, diminishing the usefulness of the WPT system.

Another thing to be considered is the recent technology trend of decreased size, weight, and power (SWaP) that the modern WPT systems should follow. Specifically, for the efficient utilization of space, modern commercial electronic devices change their form factors and are deformed, folded, and rolled, e.g., foldable, flipped phones, and rollable televisions, without degrading the performance of the electronic devices while promoting their portability. In this respect, previously demonstrated WPT systems utilizing MTMs have not been compliant with this trend, as their structures have been thick, huge, or rigid [5,6,7,8,9,10,11,12,13] for modern electronic devices. In our previous work [14], a rollable MTM screen for a high-efficiency WPT system was designed at 4.5 MHz, where only the MTM screen was rollable, while the source, Tx, Rx, and load coils were made of non-planar and non-rollable rigid structures. This implies that the Tx and Rx coils still occupied space when they were not in operation. In addition, the size of the WPT system was quite big, with an operating frequency of 4.5 MHz, being less practical and not being compliant with a standard frequency such as 6.78 MHz (AirFuel Alliance standard frequency).

In order to address these issues, we first report a high-efficiency rollable MS-based WPT. The objectives of the proposed work are as follows: (1) Achieve a high PTE of the WPT system by introducing the rollable MS screens. (2) Design a compact, planar, and rollable WPT system which occupies minimum space.

**Figure 1:**
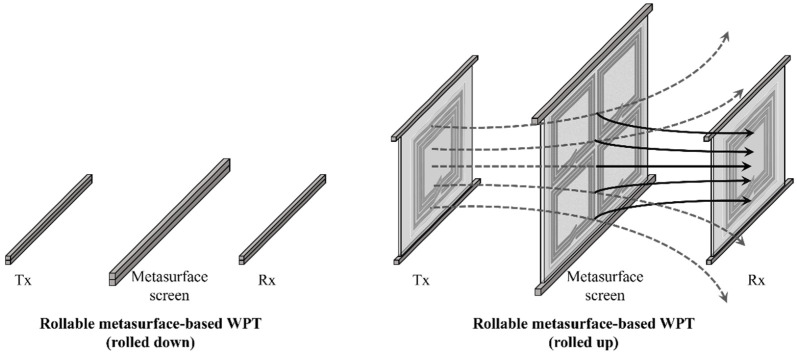
Conceptual illustration of the rollable MS-based WPT system.

As depicted in Figure 1, the proposed rollable MS-based WPT is composed of Tx, Rx, and MS screen. The rollable MS screen is located between the Tx and Rx to magnify the PTE of the WPT system. The contributions of this work are as follows: (1) By fabricating the Tx, Rx, and MS screens on a thin and flexible substrate layer, the WPT system can be rolled down or rolled up as required, which allows end users to use space more flexibly. In particular, in contrast to the traditional four-coil WPT, the source (load) coil and Tx (Rx) coil are realized in the same plane, which expands its usability and flexibility. Therefore, the compact, planar, and rollable WPT system can be realized. (2) By inserting the multiple rollable MS screens which do not require much additional space in a closed rolled-down form, the PTE of the WPT can be reconfigurably magnified without utilizing permanently located Tx/Rx and MS screens. Furthermore, we investigate the PTEs of the WPT system with multiple MS screens (up to four). The utilization of the multiple MS screens allows the WPT system to achieve the high PTE.

This article is organized as follows: Section 2 introduces the design and analysis of the rollable MS-based WPT system which include in-depth theoretical and simulation analysis. Then, the measurement results will be discussed in Section 3. The PTE measurement of the rollable WPT with multiple MS screens; the PTE measurement of the misaligned rollable MS-based WPT; and the comprehensive comparison of the rollable MS-based WPT system with previously demonstrated MTM or MS-based WPT systems, will be reported. Finally, the conclusion will be drawn.

## 2. Design and Analysis of the Rollable MS-Based WPT System

In this section, the rollable MS-based WPT system is described and analyzed. In the MS-based WPT design, fully planar and rollable structures were considered when designing the Tx/Rx and MS screens to address the limitations associated with the volumetric geometry of nonplanar structures.

### 2.1. Design and Analysis of the Rollable WPT

**Figure 2:**
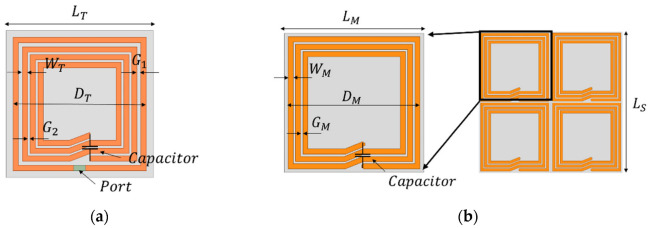
Configuration of: (**a**) Tx/Rx coils, **(b)** MS unit cell (left) and MS screen consisting of 2 × 2 unit cells (right).

**Figure 3:**
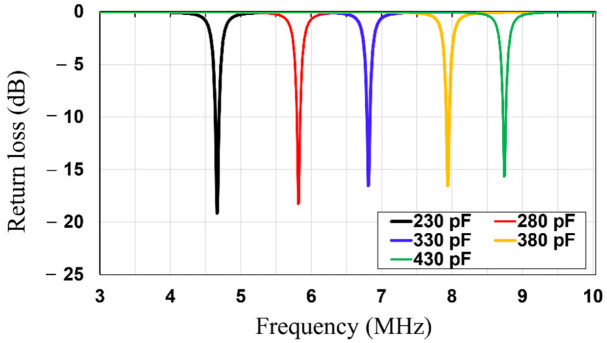
Simulated return losses of the Tx/Rx with the various capacitor values.

**Figure 4:**
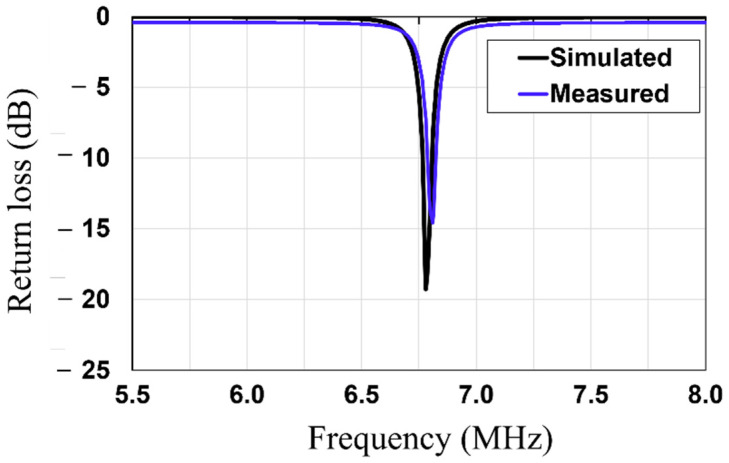
Simulated and measured return losses of the Tx/Rx coils.

First, the rollable WPT system (Tx/Rx) was engineered as depicted in Figure 2a. The proposed WPT is composed of the Tx and the Rx coils which were fabricated on a thin and flexible polyethylene ($\varepsilon_{r}=2.25)$. The thicknesses of the polyethylene substrate and copper were 0.076 mm and 0.079 mm, respectively. The thickness of the copper layer is thicker than the three skin depths of the copper at 6.78 MHz. As the rollable WPT system has thin substrate and conductor layers, it is flexible and easy to roll. In addition, in order to realize the planar Tx and Rx structures, both the Tx and Rx coils were engineered with the utilization of a 3-turn spiral and single-turn loop on the same side of the substrate. The single-turn loop acts as source/load coils and the inner 3-turn spiral acts as Tx/Rx coils of the traditional 4-coil WPT system. Table 1 shows the dimensions of the Tx, Rx, and MS unit cell. The lumped capacitors (330 pF) were parallelly connected to Tx/Rx elements to realize the magnetic resonance coupling WPT at 6.78 MHz. High Frequency Structure Simulator (HFSS, Ansys Inc., Canonsburg, PA, USA) was used to simulate and fine tune the designed WPT. After the dimensions of the Tx/Rx were designed, the value of the capacitor was swept from 230 to 430 pF, as shown in Figure 3. Based on the results, a 330 pF capacitor was chosen to be connected to Tx/Rx. Figure 4 depicts the simulated and measured return losses of the Tx and Rx with connected capacitor. It is shown that the simulated and measured return losses match very well.

### 2.2. Design and Analysis of the Rollable MS Screen

**Figure 5:**
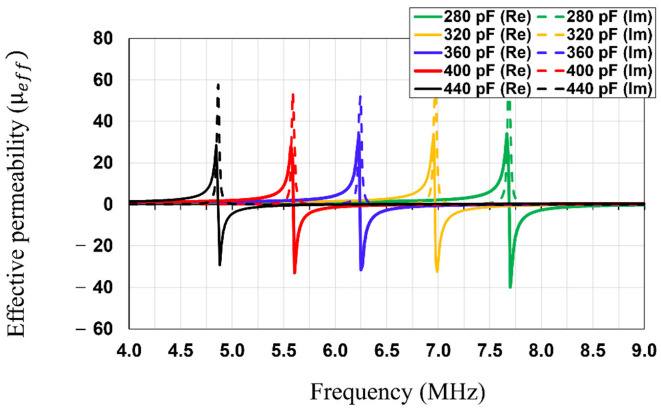
Simulated effective magnetic permeability of the MS screen with the various capacitor values.

**Figure 6:**
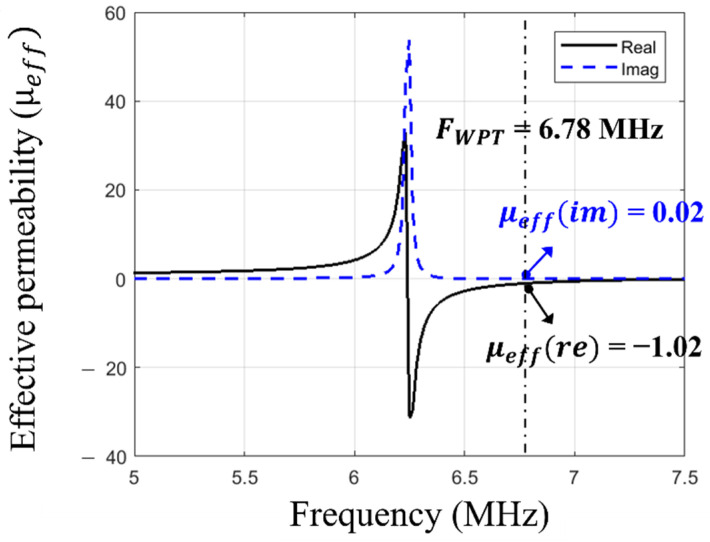
Simulated effective magnetic permeability of the MS screen.

As for the rollable MS-unit cell design, a square spiral (3-turns) was selected because it has a comparably higher quality factor than that of a split ring resonator (SRR) structure [15]. As depicted in Figure 2b, the MS screen is composed of 2 × 2 unit cells. The number of unit cells highly depends on the dimension of the WPT system. Once the dimension of the MS unit cell is determined, the number of the unit cells should be determined based on the coil size (Tx/Rx). If the entire MS screen size is smaller than the coil size (e.g., 1 × 1), the focused area of the magnetic flux becomes too small compared to the coil size, which does not take advantage of the MS screen. On the other hand, if the MS screen is significantly bigger than the coil (e.g., 3 × 3), the magnetic flux coverage could be extended. However, the loss of the MS screen also increases due to the increased number of MS unit cells [6]. Therefore, we concluded that the optimal number of the MS unit cell for the rollable WPT was 2 × 2. The MS unit cell was engineered using the same substrate and metal layers as Tx/Rx, thereby achieving its rollability. In order to realize the negative refraction property at the operating frequency (6.78 MHz), a 360 pF capacitor was connected parallelly to each unit cell. As depicted in Figure 5 and Figure 6, the negative refraction property was simulated using HFSS in order to validate its beam-focusing property. The effective magnetic permeability and refraction index were extracted from the simulated results by applying the following retrieval methods [16,17,18].

(1) $$z=\pm \sqrt{\frac{{\left(1+S_{11}\right)}^{2}-S_{21}{}^{2}}{{\left(1-S_{11}\right)}^{2}-S_{21}{}^{2}}}\;\;\;$$

(2) $$n_{eff}=\frac{1}{k_{0}d}\{\left[{\left[\ln\left(e^{ink_{0}d}\right)\right]}^{\prime \prime }+2m\pi \right]-i{\left[\ln\left(e^{ink_{0}d}\right)\right]}^{\prime }\}$$

(3) $$e^{ink_{0}d}=\frac{S_{21}}{1-S_{11}\frac{z-1}{z+1}}$$

(4) $$\mu_{eff}=n_{eff}z$$

where z is the impedance; S11 and S21 are the reflection and transmission coefficients; d is the thickness of the screen at its thickest spot; ${\left(\cdot \right)}^{\prime }$ and ${\left(\cdot \right)}^{\prime \prime }$ indicate the real part and imaginary part of the complex numbers; $n_{eff}$ is the effective refractive index;$\;k_{0}$ is the wavenumber; m is the integer correlated with the branch index of $n^{\prime }$; and $\mu_{eff}$ is the effective magnetic permeability. The real part of the effective permeability reflects the refractive index, which implies the direction of the magnetic field by the boundary conditions, and the imaginary part represents the magnetic loss [5]. The desired effective refractive index value was carefully designed by sweeping the value of the connected capacitor in HFSS, as shown in Figure 5. The capacitance value of 360 pF was determined to achieve the negative refractive index at 6.78 MHz. In the simulation results (Figure 6), the real part of the effective permeability was engineered to be −1.01 at 6.78 MHz, which means it has an effective refractive index value of −1.01. The imaginary part of the effective permeability is shown to be 0.01, which shows it has a comparably low magnetic loss. The simulated results indicate that the MS screen changes the direction of the magnetic field at the boundary to negative, thereby magnifying the PTE of the WPT at 6.78 MHz.

### 2.3. Equivalent Circuit Model of the Proposed Rollable MS-Based WPT System

**Figure 7:**
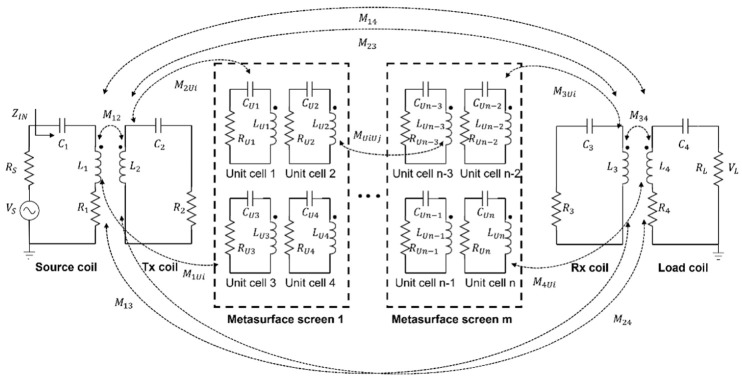
Equivalent circuit model of the rollable MS-based WPT system.

The equivalent circuit of the proposed rollable MS-based WPT system was modeled and analyzed, as depicted in Figure 7. The rollable WPT was modeled as a 4-coil WPT. In this work, multiple MS screens were added between Tx and Rx for the high PTE. The multiple MS screens can be inserted without taking up any dedicated space because of their rollability. The parameters of each element are expressed using RLC models. The system equations of the equivalent circuit can be analyzed using Kirchhoff’s voltage law (KVL), as described in Equation (5), where $V_{S}$, $I_{S}$, and $R_{S}$ are the source voltage, current, and resistance; $R_{k}$, $L_{k}$, $C_{k}$, and $I_{k}$ are the resistance, inductance, and capacitance, current of each element k $\left(k=1,\;2,\;\ldots ,\;4\;and\;U_{1},\;U_{2},\ldots ,\;U_{n}\right);\mathrm{and}\;M_{xy}\;\left(x\neq y\right)$ is the mutual coupling between two elements x and y $\left(x,\;y=1,\;2,\;\ldots ,\;4\;and\;U_{1},\;U_{2},\ldots ,\;U_{n}\right)$.


(5) $$\left\{ \begin{array}{c} \left(R_{s}+j\omega L_{1}+\frac{1}{j\omega C_{1}}+R_{1}\right)I_{s}+j\omega M_{12}I_{2}+\cdots +j\omega M_{1Ui}I_{Ui}+\cdots +j\omega M_{13}I_{3}+j\omega M_{14}I_{4}=V_{S}\\ j\omega M_{12}I_{1}+\left(R_{2}+j\omega L_{1}+\frac{1}{j\omega C_{1}}\right)I_{2}+\cdots +j\omega M_{2Ui}I_{Ui}+\cdots +j\omega M_{23}I_{3}+j\omega M_{24}I_{4}=0\\ j\omega M_{1i}I_{1}+j\omega M_{2i}I_{2}+\cdots \left(R_{Ui}+j\omega L_{Ui}+\frac{1}{j\omega C_{Ui}}\right)I_{Ui}+j\omega M_{UiUj}I_{Uj}+\cdots +j\omega M_{Ui3}I_{3}+j\omega M_{Ui4}I_{4}=0\\ \;j\omega M_{13}I_{1}+j\omega M_{23}I_{2}+\cdots +j\omega M_{Ui3}I_{Ui}+\cdots +\left(R_{3}+j\omega L_{3}+\frac{1}{j\omega C_{3}}\right)I_{3}+j\omega M_{34}I_{4}=0\\ \omega M_{14}I_{1}+j\omega M_{24}I_{2}+\cdots +j\omega M_{Ui4}I_{Ui}+\cdots +j\omega M_{34}I_{4}+\left(R_{4}+j\omega L_{4}+\frac{1}{j\omega C_{4}}+R_{L}\right)I_{4}=0 \end{array}\right.$$

In the circuit model, the transmission coefficient S21 can be used to evaluate the power-transfer capability of the circuit. S21 can be described as Equation (6) [19]:

(6) $$S_{21}=2\frac{V_{L}}{V_{S}}{\left(\frac{R_{S}}{R_{L}}\right)}^{1/2}\;\;$$

### 2.4. Magnetic Field Distribution Analysis of the Rollable MS-Based WPT System

**Figure 8:**
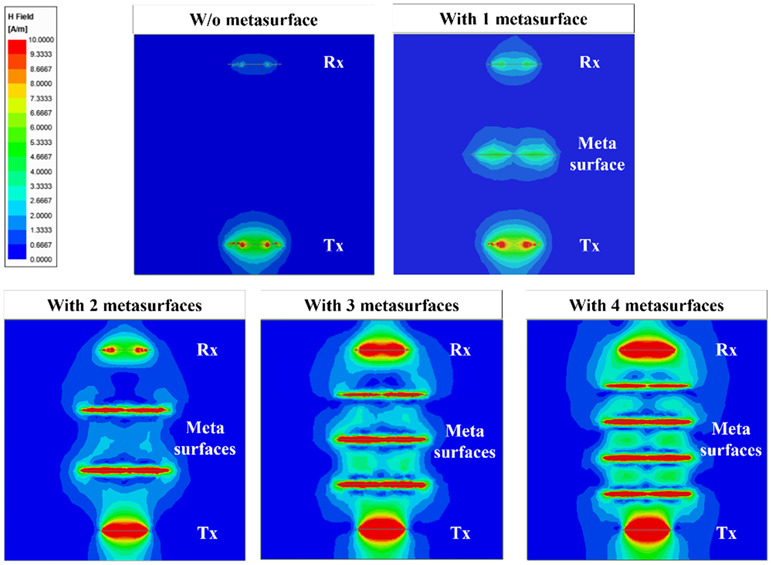
Comparison of magnetic-field distribution between the Tx and Rx without and with the MS screens.

In this subsection, the magnetic field distributions between Tx and Rx without and with MS screens are simulated at a PTD of 60 cm (Figure 8), to investigate the effect of the rollable MS screens on the magnetic energy received by the Rx element. It is observed that the magnetic-field strength around the Rx element and the magnetic coupling between Tx and Rx are greatly magnified when the MS screens are inserted. This results in S_21_ enhancement of the WPT system owing to the increased *V_L_*. Furthermore, as the number of MS screens increases, the magnetic-field intensity near the Rx increases, which proves the usefulness of the rollable MS screens.

**Figure 9:**
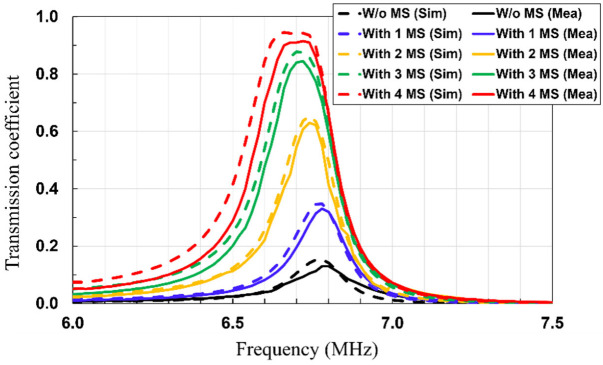
Simulated and measured transmission coefficient (S_21_) of the rollable MS-based WPT system without and with MS screens at a PTD of 60 cm.

In addition, the transmission coefficients (S_21_) of the WPT system without and with MS screens are simulated and measured at a PTD of 60 cm (Figure 9). As shown in Figure 9, the values of the transmission coefficients are greatly increased when the MS screens are inserted. These results are consistent with the ones in the magnetic-field distribution analysis. Moreover, it is observed that the resonant frequency of the rollable WPT shifts down more when the additional MS screens are inserted, as the more mutual inductance between the MS screens and Tx/Rx is induced. It is shown that the simulated and measured transmission coefficients match very well.

## 3. Measurement Results

**Figure 10:**
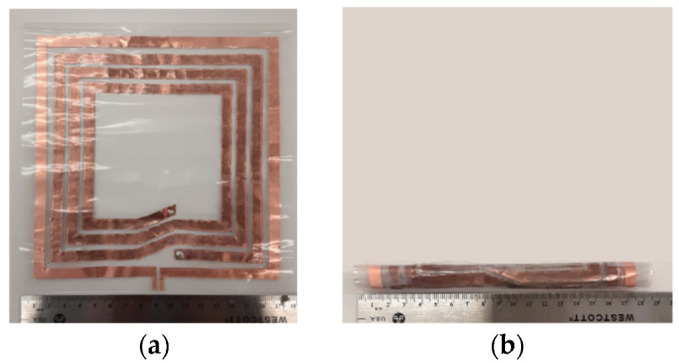

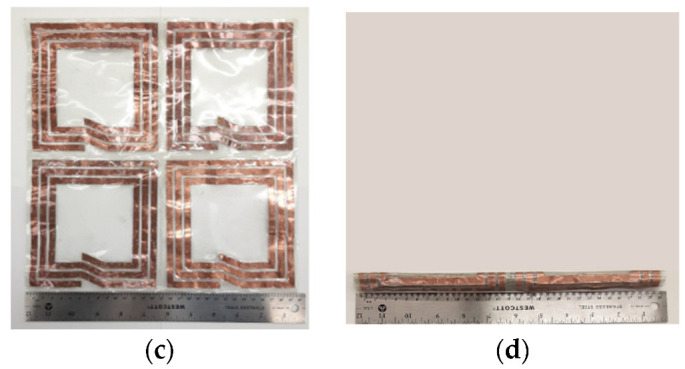
Fabricated rollable MS-based WPT system: (**a**) Tx/Rx (rolled up), (**b**) Tx/Rx (halfway rolled down), (**c**) MS screen (rolled up), (**d**) MS screen (halfway rolled down).

**Figure 11:**
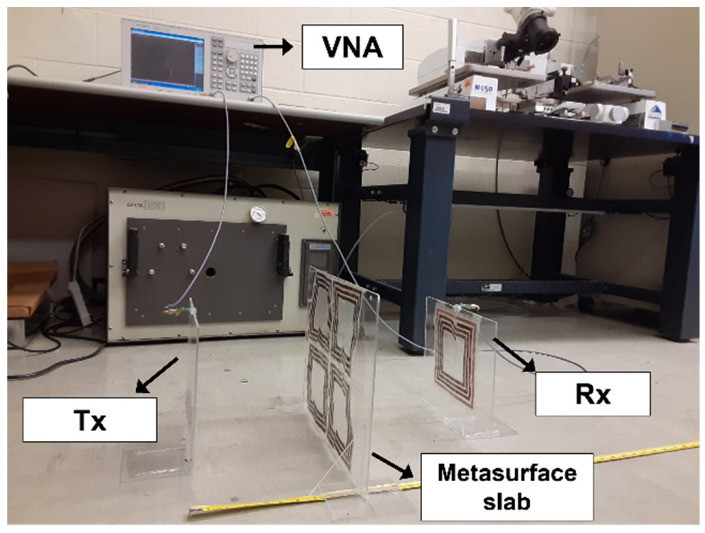
Measurement setup.

The rollable MS-based WPT system is fabricated and characterized. Figure 10 shows the fabricated rollable WPT system in the status of rolled up and rolled down (halfway) and Figure 11 shows the measurement setup. In the measurement, the Tx, Rx, and MS were fixed to the acrylic board to have flat surfaces as they are rollable and flexible. We measured the PTE utilizing a vector network analyzer (E5071C, Agilent, Inc., Santa Clara, CA, USA). The experiments were performed with a source and load impedance of 50 ohms and a source power of 10 dBm. The PTE was calculated from the measured data utilizing Equation (7) [20]:

(7) $$PTE={\left|S_{21}\right|}^{2}\times 100\;\%\;\;$$

### 3.1. Measurement of PTE

We measured and characterized the PTE of the WPT with the rollable MS screens (rolled up) and without the rollable MS screens (rolled down). The measured PTD ranges from 0 to 120 cm. In addition, the effects of the number of MS screens (up to 4) on the PTE of the WPT are studied for the first time. Since the rollable MS-based WPT system is rolled up only when charging is required, there is no need for additional space even if the additional MS screens are inserted. During measurement, the distance between inserted MS screens was maintained. The rollable MS-based WPT did not work in tune during this measurement, which means the values of the connected capacitors were designated.

**Figure 12:**
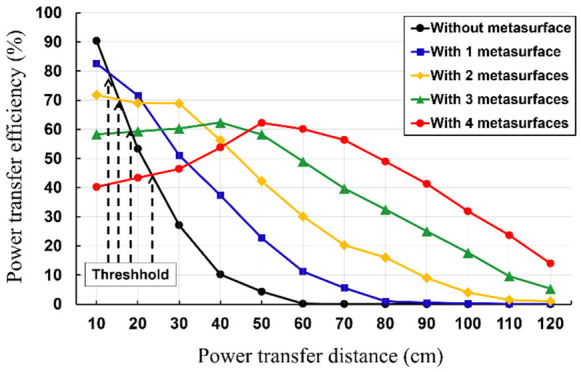
Measured PTE of the rollable WPT system without and with MS screens (up to 4).

As depicted in Figure 12 are the threshold distances, beyond which the rollable WPT with the MS screen exhibits magnified PTE. The reason for this phenomenon is that the mutual inductance between the MS screens and Tx/Rx elements increases as the PTD decreases. Hence, as the PTD decreases, the resonant frequency of the rollable WPT is shifted, thereby degrading the PTE. Moreover, it should be noted that the threshold distance increases as more MS screens are inserted. The reason for this phenomenon is that the resonant frequency of the rollable WPT shifts more when the additional MS screens are inserted, as more mutual inductance between the MS screens and Tx/Rx elements is induced. Therefore, the WPT system additional MS screens require a longer threshold point for recovering the resonant frequency of the WPT system. This also means that the optimum number of MS screens should be determined depending on the PTD. For example, the optimum number of the MS screens at the PTD of 30 cm is two as the PTEs of the WPT system with more than two MS screens are lower than that with 2 MS screens due to the shifted resonant frequency.

As for the measured results, the PTE of the rollable WPT system improves from 13.32% to 32.49% (at a PTD of 40 cm) with one MS screen, 5.42% to 42.25% (at a PTD of 50 cm) with two MS screens, 1.78% to 49% (at a PTD of 60 cm) with three MS screens, 0.85% to 46.24% (at a PTD of 70 cm) with four MS screens. In addition, the PTE of the rollable WPT system at a PTD of 60 cm improves from 1.78% (without MS screen) to 11.2% (with one MS screen), 30.09% (with two MS screens), 49% (with three MS screens), and 60.11% (with four MS screens), respectively. The measured PTE results indicate that the demonstrated rollable MS screens are significantly effective for magnifying the PTE and the PTD of the rollable WPT.

### 3.2. PTE Measurement of the Misaligned Rollable MS-Based WPT

In practical WPT applications, it is hard to achieve the perfect alignment between the Tx and Rx. The PTE of the WPT is significantly deteriorated by misaligned conditions. Hence, the WPT should consider the effects of the misalignments, and countermeasures, to recompense the decreased PTE due to the misalignments for practical WPT applications.

**Figure 13:**
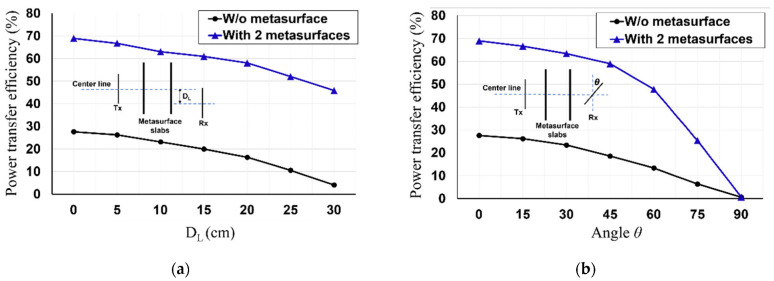
Measured PTE of the rollable MS-based WPT with: (**a**) lateral misalignment (D_L_) at 30 cm, (**b**) angular misalignment (*θ*) at 30 cm.

Here, the effects of the misalignments of the PTE of the rollable MS-based WPT are investigated. We explored the effects of the lateral and angular misalignments on the PTE of the rollable MS-based WPT without and with two MS screens at a transfer distance of 30 cm. In Figure 13a, as the misaligned lateral distance (D_L_) increases, the PTE of the rollable MS-based WPT decreases for both scenarios (without and with the MS screens). However, it is worth noting that the PTE increases significantly for the entire D_L_ when the MS screens are rolled up. Furthermore, the investigations of the angular misalignment (*θ*) are also depicted in Figure 13b. When the angle of the Rx increases, the PTE of the rollable WPT without and with the MS screens is degraded. It is shown that the impacts of the MS screens on the PTE of the rollable WPT decrease as the angular misalignment becomes worse. In particular, the magnified PTE due to the MS screens is close to 0% when the angular misalignment is 90°. This implies that the magnetic fields induced by the Tx can not be transmitted to the Rx when the misaligned angle is 90°, even if the magnetic fields are magnified and focused by the MS screens. In spite of this phenomenon, it is proved that the MS screens recompense the effects of the misalignments on the WPT.

### 3.3. Measurement Comparison

The demonstrated rollable MS-based WPT system is compared with previously demonstrated MTM- or MS-based WPT systems in Table 2. For equitable comparison, the PTD was normalized to the geometrical mean of Tx and Rx radius as described in Equation (8) [21].

(8) $$D_{\;norm}=\frac{D}{\sqrt{r_{T}\cdot r_{R}}}\;$$

where D, $D_{\;norm}$,$\;r_{T}$ and $r_{R}$ are the distance, normalized distance, Tx radius, and Rx radius, respectively. In addition, a figure of merit (FoM) was adopted to compare those MS-based WPT systems taking into account the PTE, the coil diameter, and the PTD [22]:

(9) $$FoM=D_{\;norm}\times PTE$$

The rollable MS-based WPT system has exhibited overall enhanced PTE compared to other work, while this is the only work addressing a fully planar rollable MS-based WPT system among the selected work. In addition, the PTE of the WPT system with multiple MS screens (up to four) was investigated for the first time. Especially, the PTE of the rollable MS-based WPT system with four MS screens exhibited an FoM of 2.42, which is the highest FoM among the selected work. Moreover, it is shown that the rollable MS-based WPT system has the thinnest Tx, Rx, and MTM structures among the selected work, thereby realizing the ultra-thin and rollable configuration of the WPT system. In contrast to the previously reported work, the source (load) coil and Tx (Rx) coil are realized in the same plane, which expands its usability and flexibility. It is verified that a fully planar, thin, rollable, and highly efficient MS-based WPT system is reported for the first time. The rollable nature of the Tx/Rx, and MS offers the flexibility and practicality of space usage in contrast to other MTM- or MS-based WPT, which occupy space permanently whether they are in use or not. In this respect, it is beneficial to introduce more MS screens for high PTE because the MS screens do not require additional space in the closed rolled-down form.

Given the aforementioned advantages, the proposed rollable MS-based WPT system can be applied to various scenarios. For example, the Rx of the WPT system can be directly applied to flexible or rollable electronics. The rollable Tx and MS screens can be rolled down, e.g., onto the table or ceiling when they are not used, and, thus, do not require any designated space. Then, end users charge the Rx with high efficiency by rolling up the Tx and MS screens only when needed. The number of MS screens can be determined depending on the application. In addition, the users can carry the rollable MS-based WPT system as it is in small size when rolled down. Then, users can set up the WPT systems and charge their devices as needed. If high-efficiency charging is required, the additional MS screens can be inserted between Tx and Rx.

## 4. Conclusions

In this work, a fully planar and rollable MS-based WPT was demonstrated for the first time. In the measured results, the PTE of the rollable WPT increases from 13.32% to 32.49% (at a PTD of 40 cm) with one MS screen, 5.42% to 42.25% (at a PTD of 50 cm) with two MS screens, 1.78% to 49% (at a PTD of 60 cm) with three MS screens, and 0.85% to 46.24% (at a PTD of 70 cm) with four MS screens. The measured PTE results indicate that the demonstrated rollable MS screens are significantly effective for magnifying the PTE and the PTD of the rollable WPT. In addition, the effects of the number of MS screens (up to four) on the PTE of the WPT system were investigated. It is worth noting that the number of MS screens can be chosen depending on the application. In addition, the measured PTE results in the misaligned condition verify that the MS screens still increase the PTE of the rollable WPT, even in the misaligned condition. It is expected that the demonstrated rollable MS-based WPT will open up new possibilities for practical WPT applications with improved PTE and PTD in various IoT environments.

## Figures and Tables

**Table 1:** Dimensions for Tx, Rx, and MS Unit Cell.

Parameter	Value (mm)
*L_T_*	180
*D_T_*	163
*W_T_*	7
*G*_1_	5
*G*_2_	3
*D_M_*	140
*L_M_*	150
*L_S_*	300
*G_M_*	3
*W_M_*	7

**Table 2:** Comparison of this work with other MTM- or MS-based WPT systems.

Ref.	Working Frequency (MHz)	$d_{T},\;d_{R}\;(\mathrm{mm})$	Property of MTM	Thickness of the Tx, Rx/MTM (mm)	Planar/Rollable	Configuration of the MTM/# of slabs	Transfer Distance (mm)	Normalized Transfer Distance	PTE with MTM (%)	Figure of Merit
[5]	27	400	$\mu_{eff}=-1$	>2/1.64	☓/☓	Double sided/2	500	1.25	47	0.59
[6]	6.5	600	$\mathrm{Negative}\;\mu_{eff}$	4/1.2	☓/☓	Single sided/2	1000	1.67	45	0.75
[7]	7.43	150	$\mu_{eff}=0,-1$	1.6/1.6	○/☓	Double sided/1	200	1.33	18.6	0.25
[8]	6.06	42	$\mathrm{Negative}\;\mu_{eff}$	1.2/1.2	○/☓	Single sided/2	27	0.64	12.27	0.08
[9]	5.57	40	$\mu_{eff}=-1$	-/26	☓/☓	3D structure/1	40	1	35	0.35
[9]	3	500	$\mu_{eff}=-1$	160/>1.6	☓/☓	Single sided/2	1200	2.4	49.63	1.19
[10]	15	110	$\mathrm{Negative}\;\mu_{eff}$	5.57/5.57	☓/☓	Single sided/1	120	1.09	72	0.78
[12]	8	700, 300	$\mu_{eff}=-0.5\;$~ 1.5	90, 300/96	☓/☓	3D structure/1	1200	2.62	52.05	1.36
[14]	4.5	600	$\mu_{eff}=-1$	3.588/0.16	☓/☓	Single sided/1	900	1.5	63.04	0.95
This work	6.78	163	$\mu_{eff}=-1$	0.16/0.16	○/○	Single sided/1	400	2.45	32.49	0.92
						Single sided/2	500	3.07	42.25	1.3
						Single sided/3	600	3.68	49	2.21
						Single sided/4	700	4.29	46.24	2.42

## Data Availability

Not applicable.
